# Is Exercise Blood Pressure Putting the Brake on Exercise Rehabilitation after Acute Type A Aortic Dissection Surgery?

**DOI:** 10.3390/jcm11102931

**Published:** 2022-05-23

**Authors:** Na Zhou, Warner M. Mampuya, Marie-Christine Iliou

**Affiliations:** 1 Department of Cardiac Rehabilitation and Secondary Prevention, Corentin Celton Hospital, 92130 Paris, France; tuaslapeche@hotmail.com; 2 Service de Cardiologie, Centre Hospitalier Universitaire de Sherbrooke, Sherbrooke, QC J1H 5H3, Canada; warner.mampuya@usherbrooke.ca

**Keywords:** type A acute aortic dissection, surgery, exercise, rehabilitation, blood pressure

## Abstract

(1) Background: Exercise is recommended to improve physical fitness in patients recovering from acute type A aortic dissection (ATAAD). However, surgery corrects the diseased blood vessels and reduces the risk of ATAAD, but it does not redefine a safe exercise blood pressure (BP) threshold. This review aimed to discuss whether the safe threshold of exercise BP can be upregulated after ATAAD surgery to increase exercise intensity with additional benefits. (2) Data sources: The PubMed databases were searched with the keywords “type A acute aortic dissection surgery”, “exercise”, “BP”, “stress”, and variations of these terms. (3) Study selection: Data from clinical trials, guidelines, and recent reviews were selected for review. (4) Results: Regular exercise can be considered a cardioprotective intervention for aortic dissection patients by attenuating hemodynamic responses at rest and during exercise. Previous studies have mainly focused on moderate-intensity aerobic exercise. In practice, the exercise systolic BP of some patients was higher than 160 mm Hg without adverse events, which indicates that the training intensity may be underestimated for patients after ATAAD surgery. Limited studies suggest a light-to-moderate resistance training for selected patients because it may cause a greater increase in BP. (5) Conclusions: Moderate-intensity continuous aerobic exercise supplemented by low-intensity resistance training is appropriate for cardiac rehabilitation after ATAAD surgery. The BP increase based on the normal exercise BP response, corresponding to the moderate-intensity is relatively safe. For high-risk post-ATAAD patients, considering the overall volume of training, personalizing the exercise regimen to remain within “safe” BP limits, and avoiding excessive fluctuations in BP should be the primary considerations for exercise training.

## 1. Brief Summary

Surgery repairs the affected portion of the aorta and changes the physiology, anatomy, and future risk factors for the patients, but it does not redefine a safe exercise blood pressure (BP) threshold and safe training intensity. Considering the overall volume of training, personalizing the exercise regimen to remain within “safe” BP limits and avoiding excessive fluctuations in BP should be the primary considerations for exercise training in post-ATAAD patients.

## 2. Introduction

High blood pressure (BP) is the main comorbidity [1] and risk factor [2,3] of acute type A aortic dissection (ATAAD) as well as the main concern for exercise rehabilitation in patients after ATAAD surgery. Indeed, BP is the primary force applied to the aorta wall and is closely related to wall mechanical stress. However, depending on the circumstances, this mechanical stress can be either protective or pathological [4].

In the case of ATAAD, surgical intervention improves the medium-term survival of patients [5,6], alters the anatomy, corrects diseased vessels, and reduces the risk of underlying disease, but does not redefine the safe BP threshold for exercise training. However, the safe threshold of exercise BP may be an important breakthrough point to improve the outcomes of exercise rehabilitation in the future. Therefore, the aim of the review is first to discuss whether the safe threshold of exercise BP could be upregulated after ATAAD surgery. Second, whether exercise intensity can be appropriately increased within safe exercise BP thresholds to obtain additional exercise benefits. This review focuses on exercise BP and exercise intensity in patients after ATAAD surgery covering pathophysiology, surgical treatment, and cardiac rehabilitation.

## 3. Literature Search Strategy

The literature search for this review was conducted using the PubMed electronic database. The following key terms were combined, using the operators ‘AND’ and ‘OR’: [aortic dissection OR type a aortic dissection OR post–aortic dissection OR aortic dissection surgery OR aortic dissection open repair] AND [exercise OR exercise training OR exercise intensity OR aerobic exercise OR resistance training OR resistance OR cardiac rehabilitation OR exercise rehabilitation OR rehabilitation OR physique activity OR fitness OR CPET OR endothelial OR graft OR pathophysiology OR physiology] AND/OR [blood pressure OR shear stress] OR [guidelines OR consensus OR management]. Articles involving conference abstracts were excluded.

## 4. ATAAD Pathophysiology

At present, there is limited knowledge about the link between the alterations to aortic mechanics (i.e., changes in geometry, material composition/properties, and hemodynamic loads) and the response to exercise before and after ATAAD surgery, especially regarding the interaction between exercise hemodynamics and the surgical graft, the remaining aortic vessels, and the junction part. It is known that, in addition to supplying blood, the aorta plays an important role in controlling systemic vascular resistance and heart rate (HR) through pressure-responsive receptors (baroreceptors) located in the ascending aorta and aortic arch. An increase in aortic pressure results in a decreased HR and reduced systemic vascular resistance, and vice versa [6]. Moreover, the aortic wall elasticity has the effect of a “second pump” (Windkessel function), which is of the utmost importance to maintain organ perfusion for all tissues, not only coronary arteries [6]. Open surgical strategies for ATAAD may affect the baroreceptors and the Windkessel function, which would significantly impair the body’s ability to regulate BP and HR, including the hemodynamic response to exercise. However, Hornsby et al. [7] observed normal HR and BP responses to cardiopulmonary exercise for patients with proximal thoracic aortic aneurysms or dissection by taking antihypertensive agents after open repair. ATAAD surgery focuses on the excision of the proximal intimal tear. The classic ATAAD surgery involves the replacement of the supra-coronary ascending aorta (or hemiarch) and the reestablishment of the flow in the distal true lumen. At least 70%, and up to 90%, of the survivors, could present a dissected distal aorta that can eventually dilate, rupture, lead to distal malperfusion or require extensive secondary interventions [8]. In this pathology, the aortic enlargement decreases the elasticity of the aorta, which limits the potential for the vessel wall to distend properly and accommodate increases in BP.

## 5. ATAAD Surgery

Expeditious surgery is the preferred approach to ATAAD in almost all instances, which significantly outperforms medical management [6,9]. The 1- and 3-year survival rates in patients with successfully surgically-treated ATAAD approximate 96% ± 2% and 91% ± 4%, respectively [10]. The strategy of intimal tear excision combined with graft replacement is considered the gold standard of life-saving treatment for ATAAD [11,12]. Aortic root replacement is recommended as Class I of recommendation and Level B of evidence in ATAAD with root aneurysm or primary entry tear in the root, and also is reasonable in patients with ATAAD and Marfan syndrome (MFS) or other hereditary thoracic aortic disorders [9].

From early-era conservative surgical strategies targeting aortic valve and root (e.g., commissural resuspension, sinus repair, and supra coronary aortic grafts) to modern-era aggressive using hemiarch and/or ascending aortic replacement with tube graft and repair or root replacement (e.g., valve-sparing root replacement, hemiarch replacement using non-covered self-expanding hybrid stent graft, thoracic endovascular aortic repair) [13,14], the 30-day cumulative incidence of reoperations on the aortic root, ascending aorta, and aortic arch decreased. The current standard approach at Stanford is an aggressive hemiarch replacement at the minimum for patients with dissection extending into the arch, which had a significantly lower unadjusted 1-year reoperation incidence on the aortic root, ascending aorta, and aortic arch (1.9%) than those who only received ascending aortic replacement (8.7%) [15].

Although the management of the aortic arch in ATAAD is under constant debate [16,17,18,19], in the modern era, the goal is to excise as much of the dissected tissue and re-establish flow in the true lumen of the aorta to avoid or reduce the lethal complications of ATAAD [20,21]. An aggressive approach to the arch and descending aorta (e.g., achieved with a supracoronary graft, replacing the dissected ascending aorta) may provide advantages such as aortic remodeling [22]. Arch surgery increased in the modern era, including any proximal aortic arch intervention or total arch replacement and frozen elephant trunk (FET). FET may reduce the late reoperation rate and provide a foundation for future stent graft placement if necessary [23,24]. However, there was no difference in survival or distal aortic reoperation in the use of composite valve graft, valve-sparing root replacement, or FET procedure between the total arch and the hemiarch replacement groups, respectively [15]. 

Given the complexity of the surgery itself, an individualized approach should be considered multifactors, such as patients’ general condition, aortic pathology, and surgeon’s preference and experience [25,26,27,28]. Replacement of the aortic root and ascending aorta with a synthetic graft solves the regional injury to the blood vessel in patients with aortic dissection (AD) or aneurysms, but it could also adversely affect the left ventricle diastolic filling and overall hemodynamics. Indeed, these synthetic grafts are significantly stiffer than the healthy ascending aorta, and they cause a local stiffening in the proximal aorta, thereby increasing the speed of the forward pulse pressure wave and central pulse pressures (i.e., systolic—diastolic pressures) [29]. As known, higher central artery stiffness and central pulse pressures can increase the likelihood of aneurysm and dissection in the pre-operative state [30]. Different pathophysiological stages of AD can exist simultaneously in one individual. However, ATAAD surgery (repair and/or replacement) only intervenes on a portion of the aortic vessel, not the complete aortic vascular, and does not change the pre-existing etiology and risk factors for the aneurysm or dissection (e.g., age, smoking, high blood pressure, atherosclerosis, the fragility of the wall due to connective tissue disorders such as MFS, hereditary thoracic aortic conditions, vasculitis or aortitis, etc.) [1,6,31]. Moreover, different surgical strategies, invasiveness of surgery, and the experience of the surgeon, might also affect the elasticity and stiffnesses of the aortic wall, such as surgically intervened junctions and un-intervened vessels. The replacement of the aortic root and ascending aorta with grafts could also increase the risk of descending aortic disease years later in MFS, suggesting that disease predilection for the proximal aorta is relative [30].

## 6. Cardiac Rehabilitation (CR)

CR provides a good platform to ensure the implementation of individualized exercise training programs through patient-centered holistic care, health education, and optimized medication treatment. ESC guidelines recommend that physical activity is advised in all patients with aortic pathology, even when the aorta is dilated or in the case of successful thoracic aorta surgery [32]. For patients with ATAAD who have received effective surgical treatment and are well tolerated, early CR is recommended as a Class IIa recommendation and Level C evidence by the Sport-Prevention Rehabilitation Exercise Group of the French Society of Cardiology [33]. Recent European and American guidelines recommend appropriate exercise training but do not redefine the evidence-based medicine evidence for CR in patients after ATAAD surgery.

Hypertension is the main risk factor for postoperative death in patients with AD [34]. ESC Guidelines recommend that the BP should be lowered to 130/80 mm Hg [6]. However, there is currently no evidence to determine the safety ceiling of exercise BP at which the patients after ATAAD surgery can achieve the best benefit from the exercise rehabilitation program.

Thoracic aortopathy, especially aneurysm, dissection, and rupture, is responsible for significant morbidity and mortality. Although the key biomechanical mechanisms and histopathological characteristics of thoracic aortic aneurysms, anatomy, and rupture seem to be different because their clinical manifestations often overlap, we tend to consider them at the same time [30].

### 6.1. Blood Pressure and Exercise Rehabilitation

BP is the main force applied to the aortic wall, and its elevation increases the mechanical stress within the wall, causing elicit cell-mediated mechanobiological responses. However, depending on circumstances, this mechanism could play a protective or pathological role [4]. On the pathological side, it can lead to, initiate, and propagate the AD [35,36]. Arterial hypertension is one of the main risk factors for AD, as direct parietal wall stress and an indirect proinflammatory trigger, leading to the excessive extracellular matrix degeneration and culminates in AD [34]. Additionally, the cyclic temporal fluctuations in intraluminal aortic pressure can contribute to dissection propagation [36]. Similarly, Humphrey et al. [30] explained that increased in-plane shear stress within the ascending aorta resulting from both increased circumferential stress from central pulse pressures, and reduced axial stress, due to cardiac dysfunction (i.e., axial unloading) could potentially exacerbate the dissection risk. The initiation of dissection is influenced principally by mean and maximum systolic blood pressure (SBP) [2], whereas the propagation of the dissection is influenced principally by elevated pulse pressure and HR. These two cardiodynamic variables are driven by the amount and pace of the stroke volume and ventricular pressure, where the contractility and relaxation are measured by the derivative of pressure over time (dP/dt) [2]. During dynamic exercise, the blood flow in the ascending aorta and aortic arch is increased by more than three-fold, and even up to six-fold, and the central pulse pressure rises by 40–55% from its resting condition [10]. In individuals without aortopathy, the increase in central pulse wave velocity during exercise is consistent with the increase in arterial stiffness [10].

Regular aerobic exercise is beneficial to lower BP [37]. In particular, dynamic exercises can lower resting BP in both normotensive and hypertensive patients [38]. Approximately 80% of patients with AD have arterial hypertension [34], therefore, this type of patient may obtain the same benefit from lowering resting BP due to exercise training. The chronic hypotensive effect following exercise training is caused by a multitude of factors such as a reduction in cardiac output, HR, and vascular resistance. It may be associated with (i) decreased peripheral vascular resistance [39], (ii) increased aerobic fitness [40], (iii) decreased insulin resistance [37,41], (iv) lowered plasma norepinephrine levels, (v) improved autonomic function [37,42], (vi) reduced visceral fat [43], and (vii) improved endothelial function [37]. Decreased arterial stiffness, sympathetic nerve discharge, and increased arterial baroreceptor sensitivity are also likely contributors to the reduction in vascular resistance [44]. An international registry of acute AD data reported by Chaddha et al. [45] showed that 36 months of aerobic exercise training (≥2 times/week) significantly reduced resting SBP. Lowering BP can reduce mechanical wall stress, thereby preventing aneurysm expansion and potentially catastrophic mechanical failure [3]. A systematic review and meta-analysis of the effects of exercise training on BP [37,46] pointed out that endurance, dynamic resistance, and isometric resistance training lower SBP and diastolic blood pressure (DBP), whereas combined training lowers only DBP. Isometric resistance training may have the largest potential for the reducing SBP. The classification of the risk to perform exercise rehabilitation (e.g., basic pathological, comorbidities, surgical procedures, complications, resting BP control, exercise BP response, etc.) should be used as the basis for defining the ceiling of individual exercise BP [32].

No study has reported the effect of long-term exercise rehabilitation on lowering resting BP in patients after ATAAD surgery, which requires more attention in future research.

### 6.2. Blood Pressure and Exercise Intensity

SBP increases linearly with the workload increase during exercise [47], and by approximately 10 ± 2 mm Hg per metabolic in healthy adults [48,49].

Surgery corrects the diseased blood vessels and reduces the risk of ATAAD. However, in practice, the exercise intensity is often restricted due to concerns about exercise BP.

#### 6.2.1. Blood Pressure and Aerobic Exercise Intensity

Schachner et al. [50] reviewed 91 patients who underwent ATAAD surgery and found that 87% reported one or more sports activities, and 82% had exercise habits prior to ATAAD. The most common exercise types were walking (51%), cycling (29%), hiking (15%), and gymnastics (14%). Considering these numbers, exercise rehabilitation intervention can bring further benefits to patients after ATAAD surgery. However, the current recommendations on which exercise regimen to implement are vague. The lack of guidelines is concerning since different types and intensities of exercise can lead to very different outcomes. Appropriate exercise interventions can protect against further cardiac events, but strenuous activity in unaccustomed patients risks provoking acute cardiac events, including AD [51].

Regular exercise, as a cardioprotective intervention for patients with AD, can improve clinical outcomes by attenuating hemodynamic responses (i.e., HR and SBP) at rest and during exercise [52]. Although the American Heart Association scientific statement on the standards for exercise testing [53] indicates that the criteria to terminate exercise testing is a BP over 250 mm Hg/115 mm Hg, a more conservative threshold defined as a peak SBP of >210 mm Hg for men and >190 mm Hg for women should be considered [7,54]. For most patients with aortic aneurysm (abdominal and thoracic aneurysm), aerobic training should be performed at an intensity of SBP < 180 mm Hg, and for patients who are at high risk of dissection or rupture (e.g., women and patients with large aneurysms), a threshold of <160 mm Hg should be used [10]. Exercise training may also reduce the rate of aneurysm expansion [10].

Norton et al. [55] recommend mild-to-moderate aerobic exercise based on exercise tolerance and BP response assessed by cardio-pulmonary exercise testing (CPET). Results from Corone et al. [56] as well as Fuglsang et al. [57] support moderate-intensity aerobic exercise intervention in the postoperative rehabilitation of ATAAD patients to improve the coronary flow reserve and quality of life. The risks associated with moderate-intensity aerobic exercise seem limited because, while it causes a slight increase in SBP, DBP remains stable [58]. The study by Mas-Stachurska et al. [59] in a transgenic MFS mouse model observed that moderate dynamic exercise mitigated the progression of the aortic root dilation and led to cardiac hypertrophy regression [59]. In humans, aerobic exercise produces only a modest increase in BP (140–160 mm Hg) in healthy individuals [60]. The BP fluctuates between 140–180 mm Hg at submaximal levels and generally does not exceed 210 mm Hg even with maximal aerobic exercise [61]. Chaddha et al. [51] recommended age-adjusted light-to-moderate dynamic exercise for most post-AD patients. This type of exercise includes comfortable to brisk walking or cycling at a perceived exertion of “fairly light” to “somewhat hard”, which is equivalent to 35–69% of the maximum HR or around 3.0–5.0 metabolic equivalents (for a middle-aged population), or 30–69% of maximal voluntary contraction. However, there are no current data about the effect of exercise training on the blood vessels of patients with ATAAD after surgical repair.

We have noticed that the average exercise systolic BP during CPET can be greater than 180 mm Hg at sub-maximal exercise level in patients after ATAAD surgery without any adverse events [57]. In the study by Corone et al. [56], 25% of patients after ATAAD surgery had an exercise SBP > 170 mm Hg. Moreover, their actual SBP may have exceeded the reported values since BP was not measured continuously. It is therefore impossible to know the exact BP in the interval between measurements. However, these limited exercise studies reviewed in this paper suggest that BP above the commonly reported threshold did not result in adverse events. There are no supporting data to prove that the change in SBP is related to the prognosis of postoperative ATAAD, and the cut-off value for this parameter cannot be defined without previously-validated maximal SBP or mean BP values. Therefore, keeping exercise BP below an acceptable threshold and avoiding excessive fluctuations in BP are the primary considerations for these patients during exercise [62]. Additional studies are required to fully validate a safe exercise BP threshold, and the change in SBP will also need to be considered.

Table 1 summarizes the studies related to exercise testing or training after ATAAD surgery, in which the exercise recommendations mainly focus on moderate-intensity aerobic exercise. There is not enough evidence to show that moderate-intensity exercise is better than low-intensity aerobic exercise. Based on the HR target calculated by the Karvonen formula for moderate-intensity exercise, Delsart et al. [63] indicated that the SBP and DBP at the ventilatory threshold were around 151 ± 20 and 77 ± 13 mm Hg, respectively. In these clinical studies, the exercise SBP of some patients was higher than 160 mm Hg, and the training BP termination criteria were not limited to 160 mm Hg. From this point of view, moderate exercise is appropriate but seems to underestimate the training intensity for this type of patient.

The volume of training, which reflects the overall energy expenditure in kilocalories, can also be tailored to the individual by adjusting exercise intensity, duration, and frequency. Therefore, when concerns about BP or other potential risks limit the intensity, it is possible to adjust the other two components of the exercise prescription in a way that is adequate for each patient, to maximize the benefits.

#### 6.2.2. Blood Pressure and Muscle Training Intensity

Aerobic endurance training is the preferred type of exercise for the rehabilitation of ATAAD survivors, but it is ideally supplemented with dynamic resistance exercise [64]. Peripheral muscle deconditioning is one of the main factors limiting the improvement of aerobic capacities in post-AD patients [63]. The term “muscle training” encompasses a broad range of exercise types and intensities, such as calisthenics, strength training, and resistance exercise. Recent studies suggest that isometric handgrip training may become a new tool in the nonpharmacological treatment of high BP [65]. However, most sports have both dynamic and static components that must be tailored independently to lower the risk of aortic events [66]. For example, maximum static exercises should be avoided because of the temporary increase in BP to around 320/250 mm Hg without any beneficial lowering of the resting BP [67]. Again, we still lack supporting data in patients after ATAAD surgery to determine the ideal exercise intensity. Ehrman et al. [10] suggested moderate aerobic exercise (40–59% HR reserve or 12–14 Borg rating of perceived exertion with BP < 160–180/100–110 mm Hg) combined with low-resistance training (<40–50% of the estimated 1-repetition maximum). Furthermore, Mayerick et al. [60] recommended that patients with AD limit their lifting intensity to 50% of their body weight in the bench press or to an equivalent level of perceived exertion for other strength exercises. Chaddha et al. [51] suggested that a light-to-moderate weight training program with limited weight per set could be potentially beneficial in selected patients and should be encouraged. Furthermore, the post-AD patients need to remember to use a low amount of weight and to stop several repetitions before failure [62], this stage may lead to a greater increase in blood pressure [68]. However, participants should stop well short of volitional fatigue and avoid using the Valsalva maneuver while lifting the weights [12]. To our knowledge, as shown in Table 1, there are only two articles involving resistance training in patients after AD surgery. In the study by Corone et al. [56], 16 patients carried out isolated segmental dynamic resistance exercises, and in the study by Fuglsang et al. [57], a muscle strength training and stretching regimen was used for cardiac rehabilitation. Indeed, the challenge is to obtain a functional gain while avoiding the “overload” of the cardiovascular system, especially an “excessive” rise in arterial pressure. In day-to-day practice, facing the need for appropriate muscle training regimens, some clinicians tried calisthenics sessions and/or segmental dynamic resistance exercises combined with aerobic training to improve cardiac rehabilitation [56]. However, the impact of these regimens is difficult to quantify and compare with standard resistance training, especially when surgical intervention changes the anatomy and risk factors of ATAAD patients. Further investigation is needed to assess whether a slight aortic enlargement overtime is acceptable in regards to the benefits of low-to-moderate intensity resistance training for postoperative cardiac rehabilitation in patients with ATAAD. In all cases, the patient’s capacity for good breathing control and the management of BP must be taken into account when assessing the potential risks and benefits. Furthermore, body composition analysis (e.g., lean muscle mass) and peripheral muscle strength assessment, as well as exercise BP monitoring during resistance training, may provide more useful information for improving exercise rehabilitation training programs in type of patients.

#### 6.2.3. Exercises to Avoid

Chaddha et al. [45] reported that 70% of acute AD including 55% of ATAAD (93% underwent surgery treatment), felt that their doctor was clear in what exercises they could and could not perform, 71% wished to receive specific recommendations about which activities are likely safe and which may be unsafe, and 76% restricted the amount of weight they lifted, pushed, or pulled since their dissection. Employment and lifestyle restrictions are reasonable and aim to reduce the mechanical stress on the aorta. Common recommendations are to avoid strenuous resistance exercise (e.g., weight lifting, pushing, or straining) [69], exercise caution, minimize the lifting of heavy objects [62], avoid isometric exercises that require a Valsalva maneuver [12], avoid high-intensity non-lifting exercises [69] as well as competitive and contact sports [50], limit activities that require extreme or maximal exertion in daily life (e.g., running, sprinting, chopping wood, shoveling snow, and mowing the lawn with a nonriding or non-self- propelled mower) [62], and, finally, avoid acute emotional stress [70]. In MFS, the aortic root is stiffer (i.e., decreased aortic distensibility and elastic properties) than in healthy individuals, which may increase the risk of aortic rupture or dissection [71,72], and ATAAD has a higher proportion. For acute AD survivors, based on Laplace’s law, strenuous exertion may promote further aortic complications by acutely increasing BP in the context of a weakened aortic wall [45]. Therefore, sports requiring a high static demand are thought to be associated with a risk of triggering acute AD [51]. For patients who wish to perform vigorous aerobic exercise (i.e., running or basketball), a symptom-limited stress test can help determine whether they have a hypertensive response to exercise or not [12]. In all cases, interval aerobic training is not recommended, even in the hospital setting [73]. In summary, the guiding principle of exercise testing and training is to avoid complications and adverse events. Therefore, high-risk exercises and actions which may lead to sudden increases in BP after ATAAD surgery should be avoided.

### 6.3. Medication and Education

Medication cannot substitute surgery, but it is crucial to stabilize the initial state of ATAAD patients and continue throughout the entire follow-up treatment [9]. Furthermore, medication and education are the cornerstones of good outcomes after ATAAD treatment, especially in patients that want to exercise. 

Medical therapy should be directed at decreasing excessive shear stress on the dissected layers of the affected aorta, thereby reducing false lumen propagation [9]. Good hemodynamic management is important after ATAAD surgery, but the choice of agents to achieve this is relatively unimportant [36]. In clinical practice, we primarily focus on the benefits of BP control for AD. β-blockers are the most common type of medication used to achieve this. They are beneficial in reducing the rate of aortic dilatation and have a long-term protective effect in patients after surgical repair of ATAAD with a cumulative dosage effect, thus improving survival [74]. The negative inotropic effects of β-blockers decrease the myocardial pressure head generated during the ventricular systole, which will decrease the systolic, mean, and aortic pulse pressure, with a lesser effect on diastolic aortic pressure [2]. Concurrently, the negative chronotropic effects will lower the HR [2]. These effects combine to decrease the normal and shear stress damage to the vascular wall [10] by reducing the intraluminal pressure and the pulse pressure as “anti-impulse” therapy [36]. On the downside, the chronotropic incompetence possibly caused by β-blockers may limit the aerobic capacity in patients who suffer from AD and who may already present with significant peripheral deconditioning [63]. The arterial vasodilators decrease the systemic arterial bed resistance, which can reduce the diastolic and mean BP with little effect on the SBP, resulting in an increased aortic pulse pressure [2]. Therefore, vasodilator therapy should not be initiated prior to rate control to avoid associated reflex tachycardia that may increase aortic wall stress, leading to propagation or expansion of a thoracic AD. Angiotensin-converting enzyme inhibitors or angiotensin receptor blockers may offer additional benefits to limit aortic root dilation over time [6,75]. Statins were proposed to mitigate aortic aneurysm growth through their pleiotropic effects and their reduction in oxidative stress [76]. 

Alterations in the lifestyle and emotional state are frequent in acute AD survivors [45]. In addition to deconditioning, the fear to exercise and concerns may contribute to reduced cardiopulmonary capacity and/or quality of life in post-AD [45,55,63]. Mayerick et al. [60] reported a causal relationship between severe emotional stress, extreme exertion, and ATAAD. In contrast, appropriate exercise can have a positive impact on cognition and emotions, thereby improving mental health by reducing the effects of stress, anxiety, and depression [77]. Therefore, helping patients with AD understand how to manage their emotions and stress is beneficial for their long-term survival. The main goal of therapeutic health education is to improve patients’ intrinsic motivation and promote changes to more healthy lifestyle habits, self-monitoring management, and compliance. 

## 7. Conclusions

Surgical treatment reduces the risk of ATAAD patients, but the safety threshold of postoperative exercise blood pressure has not been redefined, limiting the up-regulation of exercise intensity. Current data are limited and insufficient to redefine safe exercise BP thresholds. However, previous studies reported very few adverse events above conservative, safe exercise BP thresholds during exercise stress testing and exercise training, and the animal models have shown exercise benefits. Based on these previous experiences, we have a tendency to support the up-regulation of safe exercise BP thresholds to increase the individual exercise intensity appropriately, but it should be performed in experienced CR centers. The previous studies showed that the BP increase based on the normal exercise BP response, corresponding to the moderate-intensity, is relatively safe. Moderate-intensity continuous aerobic exercise supplemented by low-intensity resistance training is appropriate for cardiac rehabilitation after ATAAD surgery. For high-risk post-ATAAD patients, considering the overall volume of training, personalizing the exercise regimen to remain within safe exercise BP limits, and avoiding excessive fluctuations in BP should be the primary considerations for exercise training. Medication and education are additional tools to improve BP management at rest and during exercise to maximize the benefits derived from exercise rehabilitation.

There are currently very limited follow-up data on long-term exercise rehabilitation interventions in patients after ATAAD surgery (e.g., the changes in resting and exercise BP and BP difference, daily structural and non-structural physical activity data, pathophysiology, autopsy results, etc.). Future directions should be targeted at the study of blood pressure thresholds for safe exercise based on vascular pathophysiological changes, including histopathology, to provide more detailed information for clinical exercise rehabilitation decisions.

## Figures and Tables

**Table 1:** Studies related to exercise testing or training after ATAAD surgery.

References (Year)	Population (Type, Number, Men, Age)	CR Period	Test	CR Program or Physical Activity	Intensity	Comments
Norton, 2021 [55]	ATAAD and proximal thoracic aortic aneurysm post-surgery. ATAAD: 21, 86%, 55 (47, 59) yr	3 and 15 months	Maximal CPET on treadmill Echocardiography CTA 6-min walk testing Questionnaires (Health-related quality of life + anxiety + physical activity, etc.)	Yes	Low-to-moderate-intensity aerobic exercise	Cardiorespiratory fitness among the ATAAD group remained 36% below predicted normative values > 1 year after surgery. Suggesting CPET to assess exercise tolerance and BP response to determine whether mild-to-moderate exercise. Patients are able to perform incremental exercise without serious adverse events. 37% of patients reported moderate-to-severe anxiety at the early timepoint compared to 16% at the late timepoint.
Hornsby, 2020 [7]	ATAAD or thoracic aortic aneurysm post-surgery. ATAAD: 28, 86%, 52 (44, 60) yr	None	Maximal CPET on treadmill 2.9 months (1.8–3.5 months)	None	Moderate intensity (3–5 METS)	VO_2_peak in patients with post-ATAAD was 34% below normative values. Normal heart rate and BP responses to CPET for patients on antihypertensive argents. SBP/DBP 124/77 at rest to 160/70 mm Hg at peak. - BP termination criteria for this research study cohort were ≥180/90 mm Hg (BP likely exceeded this threshold because of the time interval between BP measurements); - Exercise termination BP criteria in the CR cohort were standard at > 240/110 mm Hg; Low risk of a major CPET event (abnormal CPET event rate of 2%).
Fuglsang, 2017 [57]	ATAAD post-surgery (6–12 weeks later): Group I (CR with maximal incremental test): 10, 60%, 56 ± 7 yr Group II (CR without maximal incremental test): 9, 67%, 64 ± 13 yr Group III (no CR): 10, 90%, 58 ± 8 yr	12 weeks	Maximal CPET on cycle-ergometer Questionnaires	Consistent aerobic activity (≥60 min*3 sessions/week); Muscle strength training; Stretching; Psychosocial support; Education.	Moderate intensity	Increases in VO_2_peak and maximal workload after CR. Training termination BP criteria: SBP > 180 mm Hg, but BP could exceed this threshold due to the time interval between measurements. SBP/DBP 143/80 at rest to 200/95 mm Hg at peak in group I. Group I had higher health-related quality of life.
Delsart, 2016 [63]	Aortic dissection post-surgery (few months later): Total: 105, 76%, 57.9 ± 12.4 yr ATAAD: 52 (47%), 58%, 54.8 ± 12.4 yr Group I (aerobic capacity alteration with VO_2_peak < 85%): 69, 78.3%, 56.2 ± 12.4 yr Group II (aerobic capacity preserved with VO_2_peak > 85%): 36, 52.3%, 61.1 ± 12.1 yr	None	Symptom-limited maximal CPET 24-h BP monitoring CTA Short Form 36 Health Survey questionnaire	None	Moderate intensity (recommend maximum workload being very close or even lower than 5 METS)	SBP/DBP at first ventilatory threshold was 151 ± 20/77 ± 13 mm Hg. Chronotropic incompetence and peripheral deconditioning were two main factors limiting aerobic capacities. Deconditioning and “fear to exercise” might underlie reduced cardiopulmonary functional capacity together with other factors. Moderate exercise should be encouraged in specialized centers.
Chaddha, 2015 [51]	Type-A and AAD post-surgery Total: 82, 30.5%, 67.8 ± 13.7 yr	None	Questionnaires	Consistent aerobic activity (≥2 sessions/week)	Low intensity	Physical inactivity increased (24%) most likely due to fear, but functional status was mostly intact, and 67% survivors of AD maintained walking exercise. Patients engaged in low-intensity aerobic activity had lower resting SBP 36 months after discharge (*p* < 0.05). Consistent practice of low- to moderate-intensity aerobic activity (3–5 METS) should be encouraged according to patients’ exercise goals. Alterations in lifestyle and emotional state are frequent in survivors of AAD. There is a possible association between depression and decreased aerobic exercise.
Corone, 2007 [56]	Type-I AAD post-surgery Total: 33, 76%, 55.1 ± 9.3 yr	44.8 ± 48 days (18 ± 10 sessions)	Incremental EST on cycle ergometer CT	Calisthenics sessions (30–45 min/session); Continuous cycling (5 min warm up + 30 min training + 5 min cooling down); Respiratory physiotherapy (>1 to 2/week); Education	Moderate intensity with 11.3 ± 1.5 on the Borg scale (‘light’)	Exercise BP < 160 mm Hg in 75% of patients. 25% of patients with exercise SBP < 150 mm Hg; 50% of patients with exercise SBP between 150–160 mm Hg; 25% of patients with exercise SBP > 170 mm Hg. Maximal workload increased (*p* < 0.05) after CR. Returned to work earlier and increased muscular strength.

Abbreviations: CR: cardiac rehabilitation; (AT)AAD: acute (type-A) aortic dissection; CPET: cardio-pulmonary exercise testing; (S/D)BP: (systolic/diastolic) blood pressure; VO_2_: oxygen consumption; EST: exercise stress test; METS: metabolic equivalents; CT(A): computed tomography (angiography); yr: year old.
